# Impact of COVID-19 on elderly population well-being: evidence from European countries

**DOI:** 10.1007/s11135-023-01656-1

**Published:** 2023-04-17

**Authors:** Gloria Polinesi, Mariateresa Ciommi, Chiara Gigliarano

**Affiliations:** 1 Department of Economics and Social Sciences, Università Politecnica delle Marche, Piazzale Martelli 8, 60121 Ancona, Italy; 2 LIUC - Università C. Cattaneo, C.so Matteotti 22, 21053 Castellanza, VA Italy

**Keywords:** Multidimensional well-being, Composite indicators, SHARE survey, COVID-19

## Abstract

The aim of this paper is to analyse the effect of COVID-19 on multidimensional well-being in the European population aged 50 and over by measuring changes in individual well-being before and after the pandemic outbreak. To capture the multidimensional nature of well-being, we consider different dimensions: economic well-being, health status, social connections and work status. We introduce new indices of change in individual well-being that measure non-directional, downward and upward movements. Individual indices are then aggregated by country and subgroup for comparison. The properties satisfied by the indices are also discussed. The empirical application is based on micro-data from waves 8 and 9 of the Survey of Health, Ageing and Retirement in Europe (SHARE), carried out for 24 European countries before the pandemic outbreak (regular survey) and in the first two years of the COVID-19 pandemic (June–August 2020 and June–August 2021). The findings suggest that employed and richer individuals suffered greater losses in well-being, while differences based on gender and education diverge from country to country. It also emerges that while the main driver of well-being changes in the first year of the pandemic was economics, the health dimension also strongly contributed to upward and downward well-being changes in the second year.

## Introduction

In recent decades, the interest in measuring well-being from both researchers and policy-makers has grown.

For many years, per capita Gross Domestic Product (GDP) has been used as a proxy for well-being in a society. However, GDP is a measure of the size of a country’s economy and does not properly reflect a nation’s welfare. For this reason, motivated by the European Commission’s ‘Going beyond GDP’ initiative^1^ and influenced by the well-known Stiglitz et al. (2009) report, scholars have recently come to agree that income is unable to capture the multi-faceted nature of well-being. Several studies have shown that well-being cannot be exclusively defined in terms of material deprivation, and that subjective aspects like the perception of the standard of living should also be considered (see for example Ivaldi et al. 2016; Bleys 2012; Noll 2002 and Sen 1980). In other worlds, since well-being is a multidimensional phenomenon, its measurement should move beyond the mere use of monetary aspects and also involve non-monetary dimensions. For instance, according to the OECD, well-being measurements should also consider jobs, housing, health, work and life-balance, education, social connections, civic engagement and governance, environment, personal security and subjective well-being (OECD 2011).

Several initiatives have arisen, which propose multidimensional well-being indicators. For instance, the Human Development Index (HDI) has been proposed by the United Nations Development Program, which is based on countries’ mean (or geometric mean) achievement in income, education and health (Malik 2013). Other important examples are the Better Life Index (BLI) established by the OECD, which aggregates achievements in 11 indicators (Durand 2015), and the Equitable and Sustainable Well-being ("Benessere Equo e Sostenibile" BES) project, resulting from a collaboration between the Italian National Institute of Statistics (ISTAT) and the National Council for Economics and Labour (CNEL). This latter is based on more than 130 indicators grouped into 12 domains.

Moreover, Rahman et al. (2005) proposed a composite index of well-being based on eight social dimensions, each including indicators for social relationships, emotions, health, work, material well-being, civil, and political liberties, personal security, and environmental quality. Pinar (2019) recently described a multidimensional well-being indicator using a generalized mean aggregation method with alternative parameters to allow for different levels of substitutability and complementarity between well-being dimensions.

After more than two years of the COVID-19 pandemic, researchers and scholars are interested in understanding its impact on individual well-being by evaluating the changes before and during the crisis.

The COVID-19 outbreak has affected the lives of people across the globe. Older adults, ethnic minorities, those with lower socioeconomic status and those with underlying health conditions have been disproportionately affected by COVID-19 (Khunti et al. 2020 and Li et al. 2020). According to VanderWeele et al. (2021), the COVID-19 pandemic has affected people’s lives in countless ways. The impact may extend to physical and mental health, social relationships, sense of meaning, identity, happiness and financial stability.

Older adults, especially those with vulnerable health conditions, have been affected even more by COVID-19 (Mueller et al. 2020). In fact, the COVID-19 pandemic has caused social disruption (e.g. job loss, social distancing, confinement), which in turn has affected individual well-being. For this reason, it is important to assess the impact the disruption of COVID-19 has on different dimensions of well-being for this vulnerable group.

Since well-being is a multidimensional concept, it is important to capture not only the economic effects of the COVID-19 crisis, but also the social ones. In fact, the unintended consequences of the decisions to contain the COVID-19 pandemic are huge and affect the well-being of Europeans in terms of economics, social relationships and health. All of Europe has experienced the largest economic recession since World War II. Due to epidemic control, social contacts have been interrupted and people avoid seeking medical treatment for fear of infection. There is broad consensus that one of the segments of the population most affected by these restrictions is the elderly, mainly due to the fact that older adults are at greater risk of becoming severely ill from COVID-19.

For these reasons, in this paper, we focus on the measurement of well-being of people that have been the most affected and vulnerable members of society during the pandemic, that is, elderly individuals. We are also motivated by Stiglitz et al. (2009), who stated that within a country, the perception of well-being may be different between older people and younger people. Thus, it could be of potential interest to measure well-being separately for different age groups. In this paper, we focus on the well-being of people aged 50 and over. This segment of the population is of particular interest if we consider that, according to Eurostat,^2^ in 2021 more than one fifth $(20.8\%)$ of the European Union population was aged 65 and over. The median age in the EU increased by 2.5 years between 2011 and 2021, rising from 41.6 years to 43.9 in 2020 and to 44.1 years in 2021. However, population ageing is not a new phenomenon but a long-term trend which began in Europe several decades ago. Consequently, the proportion of retired people is expanding.

The aim of this paper is therefore to understand and analyse the consequences of the COVID-19 outbreak on several dimensions of well-being of Europeans aged 50 and over. To achieve this goal, we use data from the Survey of Health, Ageing and Retirement in Europe (SHARE). We analyse 24 European Countries, namely Austria, Germany, Sweden, Spain, Italy, France, Denmark, Switzerland, Belgium, Czech Republic, Poland, Luxembourg, Hungary, Slovenia, Estonia, Croatia, Lithuania, Bulgaria, Cyprus, Finland, Latvia, Malta, Romania, Slovakia. Data are longitudinal and analysed at individual level, using individual sample weights.

In contrast to Grané et al. (2021), who design a shock similar to COVID-19 using SHARE data referring to wave 7 (year 2017), our analysis is based on the two most recent waves: Wave 8 Regular (October 2019–March 2020) and the two rounds of the coronavirus survey (periods June–August 2020 and June–August 2021). We propose indices of changes in well-being dimensions before and after the pandemic.

This paper looks at directional changes (upwards and downwards) of individual well-being dimensions before and after the pandemic, distinguishing between the first and second year of the pandemic.

Our approach is a novelty in the literature, since to the best of our knowledge, this is the first time that well-being at country level has been evaluated by means of individual directional changes, that is, by taking into account downward movement, upward movement, and net effect. The proposed approach bridges two streams of literature on well-being: the counting approach used in multidimensional poverty measurements (see Alkire and Foster 2011) and inter-temporal intragenerational mobility (see Gigliarano and Chelli 2016).

Here, we adopt a two-step procedure. First, individual change indices are provided, which allow for the analysis of upward, downward and overall changes in well-being due to the COVID-19 pandemic. Second, the corresponding overall indicators are introduced by aggregating the individual indices over the entire population. This second step allows for comparison among different societies.

The counting approach that we follow here entails the intuitive procedure of counting the number of indicators or dimensions for which a person satisfies a given condition. Here we consider three types of conditions: worsening, improving or unchanging a given status compared to the past. Indices will then assume values $-1$, 0 and 1, respectively, for worsened, unchanged and improved condition, as a sort of normalization procedure required in composite indicators. Another crucial step in the construction of composite indicators is the aggregation step. In this sense, the counting approach assumes a compensatory aggregation, since it implies complete substitutability across all well-being indicators (see, among others, Mazziotta and Pareto 2013, 2016, 2019). In fact, we sum the number of indicators in which an individual has improved, unchanged or worsened his condition, without considering the magnitude of this changes. This means that, due to counting approach, our indices will capture the incidence but not the intensity of changes in individual well-being.

The contribution of this paper is twofold: it offers a new perspective of the analysis of well-being by introducing three families of aggregate indices depending on a parameter that reflects sensitivity to changes. The novelty of this paper thus lies in the direction of individuals’ well-being paths by considering downward and upward movements. Moreover, it focuses on well-being for a specific group of the population, yielding an in-depth understanding of the effects of COVID-19.

We also conduct a subgroup analysis to investigate a segment of the population i.e, individuals aged 50 or more, whose well-being is more vulnerable to the COVID-19 pandemic. The findings suggest that employed (including self-employed) and richer individuals suffer greater well-being losses, due as well to a worsened work status, while the effects based on gender and education diverge from country to country.

The rest of this paper is organized as follows. Section 2 introduces the new indices of well-being change (upwards, downwards and overall deprivation) and discusses the main properties. Section 3 describes the data set used in this study. Section 4 illustrates the empirical results, revealing differences among European countries as well as among socio-demographic groups. Finally, Sect. 5 draws some conclusions.

## Methodology

The measurement of multidimensional well-being includes a variety of dimensions such as material well-being, education, health, work and social connections. In this section, we propose indices that capture changes in well-being with the aim of analysing the effects of the COVID-19 pandemic on living conditions of the population.

This paper aims to measure multidimensional well-being movements before and after the pandemic outbreak by looking at the direction of individual changes in well-being, taking into account both downward and upward movements.

We first measure individual changes in well-being, and then we aggregate the individual indices over the whole population for comparison among different societies.

### Indices for changes in individual well-being

The individual indices of well-being change compare the levels of individual well-being dimensions over two time periods (before and after the pandemic outbreak).

We therefore propose two directional measures of changes that are able to catch downward and upward movements in the individual multidimensional well-being. Moreover, we propose an index of individual well-being deprivation that measures the net effect.

Consider a population of individuals $i=1,\dots ,n$ observed in two periods of time, *t* and $t-1$. In our context, time $t-1$ indicates a pre-pandemic period of time, while time *t* refers to a period of time after the pandemic outbreak.

We assume that individual well-being is a multidimensional concept based on *K* indicators, which are quantitative or ordinal variables. We denote the individual level of well-being indicator *k* at time *t* with $x_t^{ik}$ and at time $t-1$ with $x_{t-1}^{ik}$, for $k=1,\dots , K$. Let $v_k$ be the weight of each indicator *k* such that $\sum _{k=1}^{K}v_k=1$. In our empirical analysis, we assume equal weights of the well-being indicators such that $v_k=1/K$, for $k=1,\dots ,K$. However, the approach also allows for different types of weights.

#### Definition 1

The individual index of downward well-being change is defined as:

1 $$\begin{aligned} d_i={\sum _{k=1}^{K} \mathbbm {1}(x_t^{ik} < x_{t-1}^{ik}) \cdot v_k} \end{aligned}$$

where $\mathbbm {1}(\cdot )$ is the usual indicator function.

The index $d_i$ takes values between 0 and 1. It measures the incidence of downward changes in well-being indicator for individual *i* over time. Moving from time $t-1$ to *t*, if individual *i* worsens for all well-being indicators, then his/her index is equal to 1; on the contrary, if no indicator worsens, the index is equal to 0.

#### Definition 2

Similarly, the individual index of upward well-being change is defined as:

2 $$\begin{aligned} u_i={\sum _{k=1}^{K} \mathbbm {1}(x_t^{ik} > x_{t-1}^{ik}) \cdot v_k}, \end{aligned}$$

where $\mathbbm {1}(\cdot )$ is the usual indicator function.

This index measures the incidence of improvement changes in individual well-being indicators over time. Moving from time $t-1$ to time *t*, if an individual improves in all well-being indicators, then his/her index is equal to 1; on the contrary, if no indicator improves over time the index is equal to 0.

Hence, index $d_i$ corresponds to the proportion of well-being indicators in which the condition of individual *i* worsens, while index $u_i$ corresponds to the proportion of well-being indicators in which the condition of individual *i* improves.

Therefore, both indices may be positive for an individual, for example, if he/she worsens in one or more well-being indicators while improving in one or more well-being indicators.

We therefore consider a third individual index, which is aimed at measuring the net effect of the pandemic on well-being, defined as the difference between downward changes and upward changes.

#### Definition 3

The individual index of overall deprivation can be defined as:

3 $$\begin{aligned} o_i=max\{0, d_i - u_i \}, \end{aligned}$$

where $d_i$ is the individual index of downward well-being change defined in (1) and $u_i$ is the individual index of upward well-being change defined in (2).

The index $o_i$ is equal to the difference (if positive) between the downward and upward well-being change indices; for negative differences (when the number of improvements surpasses the number of changes or the worst), the index is set equal to zero. Index $o_i$ ranges between 0 and 1 and is equal to 1 if individual *i* experiences, from time $t-1$ to time *t*, a worsening for all indicators and no improvements at all, while it is equal to 0 if the number of indicators of well-being in which individual *i* improves is greater than or equal to the number of indicators that worsened.

### Aggregate indices of well-being change

Aggregating the individual indices of changes into a synthetic measure enables an assessment of the intensity of the effects of COVID-19 on the multidimensional well-being change in a given country or group. We propose the following aggregate indices of well-being change:

- Aggregate index of downward well-being change:
  4 $$\begin{aligned} D_{\alpha }={\sum _{i=1}^{q} d_i ^{\alpha } w_i} , \end{aligned}$$
  where *q* is the number of individuals with $d_i>0$.
- Aggregate index of upward well-being change:
  5 $$\begin{aligned} U_{\alpha }={\sum _{i=1}^{p} u_i ^{\alpha } w_i} , \end{aligned}$$
  where *p* is the number of individuals with $u_i>0$.
- Aggregate index of overall well-being deprivation:
  6 $$\begin{aligned} O_{\alpha }={\sum _{i=1}^{z} o_i ^{\alpha } w_i} , \end{aligned}$$
  *z* is the number of individuals with $o_i>0$.

In each of the three aggregate indices, parameter $\alpha$ can take non-negative integer values and indicates the sensitivity to changes (in this paper we consider $\alpha =0, 1$)^3^, while $w_i$ represents the individual sample weight such that $\sum _{i=1}^{n}w_i=1$.

When $\alpha =0$, the aggregate indices correspond to headcount ratios: $D_0$ is the *downward well-being change headcount ratio* measuring the proportion of the population that has worsened with respect to at least one well-being dimension. Index $U_0$ is the *upward well-being change headcount ratio*, which measures the proportion of the population that has experienced an improvement in at least one well-being dimension. Finally, index $O_0$ is *the overall well-being deprivation headcount ratio*, which measures the proportion of the population that has experienced more changes for the worse than improvements in all well-being dimensions.

When $\alpha =1$, the aggregate index $D_1$ corresponds to *downward well-being change gap*, which measures the average proportion of well-being dimensions for which the conditions of all individuals in the society worsened. Index $U_1$ corresponds to * upward well-being change gap*, which measures the average proportion of well-being dimensions for which the condition of individuals in the society improved. Finally index $O_1$ is the *overall well-being deprivation gap* and measures the average net deprivation of the society due to the COVID-19 pandemic.

Let us discuss some of the main properties of the aggregate indices of downward and upward movements, $D_{\alpha }$ and $U_{\alpha }$.

1. *Normalization* Indices $D_{\alpha }$ and $U_{\alpha }$ are normalized, which means that their range is [0,1]. The lower bound is reached when the well-being status of no individual worsens (improves) before and after the pandemic. The upper bound is obtained when all individuals worsen (improve) with respect to all well-being dimensions before and after the pandemic.
2. *Monotonicity* All things being equal, if one individual experiences an higher number of downward (upward) changes in well-being, the aggregate indices increase.
3. *Anonymity* Any exchange among individual inter-temporal well-being profiles, by which the same changes move from one person to another, does not affect the aggregate index.
4. *Independence* Individual well-being profiles provide independent contributions to the aggregate indices.
5. *Population* *proportionality* If two or more identical populations are collected, the aggregation index does not change, i.e. the index is independent of the population size.
6. *Decomposability* The aggregate indices can be expressed as weighted means of subgroup indices, in which the weights correspond to the sizes of the groups.
7. *Subgroup* *consistency*: if well-being change increases within a given subgroup and other subgroups remain unchanged, then the aggregate indices increase.

## Data

The empirical analysis is based on data provided by the Survey of Health, Ageing and Retirement in Europe and Israel (SHARE), which is a longitudinal and interdisciplinary database gathering microlevel information on health, well-being, and socioeconomic characteristics for the population aged 50 or older in 27 countries (Europe plus Israel). Here we focus only on 24 European countries (Austria, Germany, Sweden, Spain, Italy, France, Denmark, Switzerland, Belgium, Czech Republic, Poland, Luxembourg, Hungary, Slovenia, Estonia, Croatia, Lithuania, Bulgaria, Cyprus, Finland, Latvia, Malta, Romania, Slovakia), since the Netherlands and Greece were excluded from our analysis due to a variety of missing values.

We focus on the two most recent waves of SHARE (Waves 8 and 9) and in particular on: (i) “Wave 8 Regular Survey”, which is related to the pre-COVID period and includes data collected from October 2019 to March 2020; (ii) “Wave 8 corona survey”, which refers to the first year of the COVID-19 pandemic and collected from June to August 2020; (iii) “Wave 9 Corona Survey”, referring to the second year of the COVID-19 pandemic and collected from June to August 2021,^4^

To assess the effects of COVID-19 pandemic on multidimensional well-being, we consider the following indicators of individual well-being: self-assessed health status, employment status, equivalized disposable annual income^5^ and the ability to make ends meet.^6^ In particular, Tables 1 and 2 describe the questionnaire’s items associated with the variables considered in the analysis. For more details on the data, we refer to Börsch-Supan (2017).

Moreover, to investigate the effects of the COVID-19 pandemic on specific subgroups, we also considered sociodemographic variables, such as country, gender, age, and education level (ISCED classification).

**Table 1:** Variables description, Wave 8 Regular survey versus Wave 8 Corona survey

Well-being domain	Variables	Wave 8 regular survey	Wave 8 corona survey	Question	Values
*Health*					
	csphus		X	Change in your health since the outbreak	1 Improved 2 Worsened 3 About the same
*Social connections*					
	ac035d1	X		Voluntary or charity work	1 yes 0 no
	vact		X	Volunteered since outbreak	1 yes 0 no
*Economics*					
	fdistress	X		Is your household’s total monthly income able to make ends meet?	1 With great difficulty 2 With some difficulty 3 Fairly easily 4 Easily
	cahmem		X	Since the outbreak, is your household’s total monthly income able to make ends meet?	1 With great difficulty 2 With some difficulty 3 Fairly easily 4 Easily
	thinc2	X		Overall monthly income before outbreak	numeric
	imthinc2		X	Lowest overall monthly income since outbreak	numeric
*Work*					
	cjs	X		Current job situation	1 Retired 2 Employed or self-employed 3 Unemployed 4 Permanently sick or disabled 5 Homemaker 97 Other
	unemployed		X	Work: unemployed, laid off or business closed due to COVID-19	1 yes 0 no
	empself		X	Work: employed or self-employed when COVID-19 broke out	1 yes 0 no

**Table 2:** Variables description, Wave 8 Regular survey versus Wave 9 Corona survey

Well-being domain	Variables	Wave 8 Regular survey	Wave 9 Corona survey	Question	Values
*Health*					
	sphus		X	Self-perceived health - US scale	1 Excellent 2 Very good 3 Good 4 Fair 5 Poor
	caph003		X	Rating of subjective health	1 Excellent 2 Very good 3 Good 4 Fair 5 Poor
*Social connections*					
	ac035d1	X		Voluntary or charity work	1 yes 0 no
	cas115		X	Volunteered since outbreak	1 yes 0 no
*Economics*					
	fdistress	X		Is your household’s total monthly income able to make ends meet?	1 With great difficulty 2 With some difficulty 3 Fairly easily 4 Easily
	caco107		X	Since the outbreak, is your household’s total monthly income able to make ends meet?	1 With great difficulty 2 With some difficulty 3 Fairly easily 4 Easily
	thinc2	X		Overall monthly income before outbreak	numeric
	cae107e		X	Lowest overall monthly income since last interview (July 2020)	numeric
	cae100		X	Household income same every month since last interview (July 2020)	1 yes 0 no
*Work*					
	cjs	X		Current job situation	1 Retired 2 Employed or self-employed 3 Unemployed 4 Permanently sick or disabled 5 Homemaker 97 Other
	caw102		X	Work: unemployed, laid off or business closed since last interview (July 2020)	1 yes 0 no

The original sample refers to 28014 individuals who participated in Waves 8 (Regular and Corona Surveys) and Wave 9 of SHARE. Missing values of the Wave 8 Regular Survey were assigned using information available in Wave 7, which refers to the year 2017. Missing values in the Wave 9 Corona Survey referring to the variables “lowest overall monthly income” and “ability to make ends meet”, were assigned using information in the Wave 8 Corona Survey, if the respondent states that household income remained unchanged since the last interview (July 2020) or if the respondent is retired and is not working.

After the assignment process, the percentage of missing values referring to the variable “ability to make ends meet” was 0.22% (Wave 8 Regular Survey), while missing values of the variable “lowest overall monthly income” and “ability to make ends meet” of the Wave 9 Corona Survey were $1.58\%$ and $0.57\%$, respectively.

The analysis was carried out considering two time intervals at a time: the pre-COVID situation vs the first year of pandemic (Wave 8 Regular survey vs Wave 8 Corona survey) and the pre-COVID situation vs the second year of pandemic (Wave 8 Regular survey vs Wave 9 Corona survey). We therefore consider a data set of 27674 complete cases for the comparison of the pre-COVID situation vs the first year of pandemic, and 27191 complete cases for the comparison of the pre-Covid situation vs the second year of the pandemic.

To evaluate changes in well-being between the pre-COVID period and the years of the pandemic, we first compare each individual well-being dimension before and after the pandemic outbreak. We assign values -1,0 or 1 to the change in health dimensions if the respondent affirms that his/her health status since the outbreak worsened, was about the same or improved, respectively.^7^ For changes in the work dimension, we assign a value of -1 if the respondent was working when COVID-19 broke out and lost his/her job due to the pandemic, otherwise it is 0. Note that work dimension excludes upward changes. Changes in the economic dimension depend on two specific indicators: changes in the income decile and changes in the ability to make ends meet. To assess changes in the income decile, we first construct deciles for the variables “lowest income perceived since outbreak” and “individual income perceived before the outbreak”,^8^ then we evaluate whether there are differences in the individual income position before and after COVID-19; movements to higher/lower income deciles are associated with positive (+1)/negative (− 1) changes in economic well-being. Moreover, if the ability to make ends meet improves/worsens with respect to the pre-COVID period, it implies a positive (+1)/negative (− 1) change in economic well-being.

Finally, changes in the social dimension take a value equal to − 1 if the respondent stopped voluntary work during the COVID-19 pandemic, 0 if his/her behaviour did not change, or 1 if the individual started to volunteer since the outbreak.

## Empirical findings

In this section, we present results from the empirical application of the aggregate indices of directional change in well-being and overall well-being deprivation defined in Equations (4-6) separately for the first two years of the pandemic. First, we analyse well-being changes at European level, also performing a subgroup analysis (Section  4.1). Then we investigate each country separately (Section  4.2).

As already discussed in Sect. 3, the empirical application is based on a dataset which includes respondents who participated in the SHARE Wave 8 Regular Survey, Wave 8 Corona Survey and Wave 9 Corona Survey, using the calibrated longitudinal weights available in the data.

### European evidence

To better evaluate how individual well-being changed throughout the COVID-19 pandemic, we separate the analysis, distinguishing two different comparisons: we first compare the pre-COVID situation vs the first year of pandemic (Wave 8 Regular survey vs Wave 8 Corona survey), then the pre-COVID situation vs the second year of pandemic (Wave 8 Regular survey vs Wave 9 Corona survey).

Tables 3 and 4 illustrate the values of the well-being change indices together with their confidence intervals, comparing the first and the second year of the pandemic for the overall European population as well as by subgroups (in 3 we set $\alpha =0$ and in Table 4$\alpha =1$).

Table 3a reveals that after one year of COVID-19, the percentage of the elderly European population for whom at least one well-being indicator worsened is 57.9%, while the proportion of the population that experienced an improvement in at least one well-being dimension is 57%. Moreover, the headcount of overall deprivation (i.e. the proportion of the population that experienced more changes for the worse than improvements in all well-being indicators) is equal to 37%. In the second year of COVID-19, the headcount ratio for both downward and upward changes increased significantly compared to the first pandemic period, while the net effect measured by overall deprivation decreased, reaching 35.5% (Table 3b). In particular, the percentage of individuals who experienced a worsening of well-being increased from 57.9% in the first year to 63.1% after two years. Therefore, it seems that the persistence of the pandemic sharpened the worsening of elderly well-being.

Splitting the analysis into subgroups according to gender, the differences between males and females do not seem statistically significant for any index of change considered, while education, work status and income show a significant effect. In particular, people with tertiary education experienced significantly stronger downward changes and fewer upward changes than people with primary or lower secondary education. Employed and self-employed workers were significantly more deprived than retired people (respectively, 43% vs 34% of all those deprived after one year of pandemic). Moreover, the poorest and the middle class (first, second and third income quintiles) were less affected by downward changes than individuals belonging to higher income classes (fourth and fifth quintiles). These subgroup differences appear in both years of the pandemic.

**Table 3:** Downward and upward well-being changes and overall deprivation headcount ($\alpha =0$), total and by subgroup (index and 95 % bootstrap confidence interval); first year of COVID-19 (a) and second year of COVID-19 (b)

	Downward well-being change			Upward well-being change			Overall deprivation		
	Index	95% CI		Index	95% CI		Index	95% CI	
*(a)*									
**Total**	0.579	0.569	0.589	0.570	0.560	0.579	0.370	0.360	0.380
**Gender**									
Male	0.576	0.560	0.592	0.563	0.548	0.580	0.367	0.350	0.382
Female	0.582	0.571	0.593	0.575	0.563	0.586	0.372	0.361	0.384
**Education**									
Primary-lower secondary	0.565	0.546	0.584	0.598	0.580	0.617	0.353	0.335	0.372
Upper secondary	0.584	0.570	0.597	0.574	0.559	0.589	0.372	0.357	0.385
Tertiary	0.593	0.573	0.613	0.516	0.495	0.536	0.392	0.373	0.411
**Work status**									
Retired	0.547	0.538	0.556	0.591	0.582	0.599	0.339	0.330	0.347
Employed	0.648	0.627	0.669	0.530	0.508	0.554	0.432	0.409	0.454
Other	0.542	0.513	0.570	0.583	0.554	0.614	0.341	0.311	0.370
**Income quintile**									
First	0.394	0.377	0.410	0.696	0.681	0.711	0.225	0.211	0.239
Second	0.465	0.445	0.486	0.679	0.658	0.700	0.258	0.242	0.275
Third	0.569	0.545	0.593	0.617	0.593	0.641	0.328	0.304	0.352
Fourth	0.652	0.632	0.671	0.546	0.526	0.565	0.427	0.408	0.447
Fifth	0.695	0.678	0.713	0.387	0.368	0.407	0.513	0.494	0.532
(*b*)									
**Total**	0.631	0.622	0.640	0.653	0.644	0.662	0.355	0.346	0.365
**Gender**									
Male	0.625	0.609	0.640	0.656	0.641	0.670	0.350	0.335	0.365
Female	0.636	0.625	0.646	0.651	0.640	0.663	0.360	0.348	0.371
**Education**									
Primary-lower secondary	0.633	0.618	0.649	0.690	0.675	0.705	0.339	0.322	0.356
Upper secondary	0.621	0.608	0.633	0.654	0.642	0.667	0.351	0.338	0.364
Tertiary	0.647	0.628	0.665	0.605	0.586	0.623	0.383	0.365	0.401
**Work status**									
Retired	0.612	0.604	0.620	0.676	0.668	0.684	0.327	0.320	0.336
Employed	0.675	0.652	0.697	0.613	0.590	0.636	0.407	0.383	0.431
Other	0.618	0.588	0.648	0.637	0.607	0.666	0.365	0.335	0.395
**Income quintile**									
First	0.486	0.470	0.504	0.809	0.797	0.822	0.193	0.181	0.205
Second	0.553	0.531	0.574	0.743	0.723	0.762	0.258	0.240	0.275
Third	0.596	0.575	0.617	0.698	0.679	0.718	0.323	0.300	0.344
Fourth	0.681	0.665	0.697	0.646	0.628	0.663	0.388	0.369	0.406
Fifth	0.722	0.702	0.742	0.481	0.460	0.501	0.490	0.470	0.511

Table 4 shows the values of well-being change indices when $\alpha =1$, hence corresponding to indices of change gap. The downward (upward) well-being change gap measures the average proportion of well-being indicators for which the conditions of all individuals in the society worsen (improve), while the overall deprivation gap measures the average net deprivation of the society due to the COVID-19 pandemic.

The data reveal that from the first to the second year of the pandemic, both the downward and upward change gaps increased significantly (from 16% to 18.1% and from 14.5% to 18.4%, respectively). The overall deprivation gap remained stable in the two periods, around 10.9%.

Moreover, the empirical evidence also confirms significant effects of education, work status and income classes on the change gaps, analogous to what was observed for the headcount ratio in Table 3.

**Table 4:** Downward and upward well-being changes and overall deprivation gap ($\alpha =1$), total and by subgroup (index and 95 % bootstrap confidence interval); first year of COVID-19 (a) and second year of COVID-19 (b)

	Downward well-being change			Upward well-being change			Overall deprivation		
	Index	95% CI		Index	95% CI		Index	95% CI	
(*a*)									
**Total**	0.160	0.157	0.163	0.145	0.142	0.148	0.109	0.106	0.113
**Gender**									
Male	0.158	0.152	0.162	0.144	0.139	0.149	0.108	0.103	0.113
Female	0.163	0.158	0.167	0.146	0.143	0.149	0.111	0.107	0.115
**Education**									
Primary-lower secondary	0.151	0.146	0.157	0.154	0.149	0.160	0.103	0.097	0.108
Upper secondary	0.166	0.161	0.171	0.144	0.140	0.148	0.113	0.108	0.118
Tertiary	0.163	0.156	0.169	0.132	0.125	0.139	0.113	0.107	0.119
**Work status**									
Retired	0.146	0.143	0.149	0.151	0.149	0.153	0.097	0.094	0.100
Employed	0.189	0.182	0.196	0.134	0.126	0.141	0.133	0.125	0.140
Other	0.146	0.138	0.155	0.149	0.141	0.157	0.100	0.091	0.109
**Income quintile**									
First	0.100	0.096	0.105	0.185	0.181	0.190	0.061	0.057	0.065
Second	0.120	0.114	0.126	0.179	0.173	0.186	0.074	0.069	0.080
Third	0.153	0.146	0.159	0.164	0.156	0.172	0.097	0.090	0.105
Fourth	0.185	0.179	0.192	0.133	0.128	0.138	0.127	0.120	0.133
Fifth	0.202	0.195	0.209	0.088	0.084	0.093	0.156	0.148	0.164
(*b*)									
**Total**	0.181	0.178	0.185	0.184	0.181	0.187	0.109	0.106	0.113
**Gender**									
Male	0.180	0.174	0.185	0.185	0.180	0.190	0.109	0.103	0.114
Female	0.182	0.178	0.187	0.184	0.180	0.187	0.110	0.106	0.114
**Education**									
Primary-lower secondary	0.179	0.173	0.185	0.200	0.195	0.206	0.103	0.096	0.109
Upper secondary	0.180	0.175	0.185	0.184	0.179	0.188	0.110	0.105	0.115
Tertiary	0.185	0.179	0.192	0.164	0.158	0.171	0.116	0.109	0.122
**Work status**									
Retired	0.169	0.166	0.172	0.191	0.188	0.194	0.097	0.094	0.100
Employed	0.198	0.216	0.173	0.165	0.180	0.131	0.123	0.140	0.207
Other	0.181	0.170	0.192	0.177	0.168	0.187	0.116	0.105	0.128
**Income quintile**									
First	0.126	0.121	0.131	0.247	0.242	0.252	0.054	0.050	0.058
Second	0.145	0.139	0.152	0.217	0.210	0.224	0.073	0.068	0.078
Third	0.175	0.166	0.184	0.200	0.192	0.207	0.100	0.092	0.108
Fourth	0.199	0.193	0.205	0.178	0.172	0.183	0.121	0.114	0.128
Fifth	0.215	0.207	0.223	0.123	0.117	0.129	0.154	0.147	0.162

We now analyse the joint behaviour of the changes in well-being in the two years of the pandemic. Since we observe each individual in our sample at three points of time (before the COVID-19 outbreak, in the first year and in the second year of the pandemic), we can therefore depict the dynamics of well-being changes over these periods of time. For each dimension of well-being, Table 5 shows rows indicating changes in well-being that occurred from the pre-pandemic period to the first year, while the columns refer to changes from the fist year to the second year of the pandemic.

Focusing on income, for example, the table reveals that for 32% of individuals, their income worsened both in the first and in the second year of the pandemic. For 23%, the income decile did not change over the two years, while the economic situation for 39% of individuals improved in both years.

Moreover, the key role played by self-perceived health during the second year of the COVID-19 pandemic emerges: individuals who declared no changes in their health one year after the COVID-19 outbreak, did, however, claim changes in their health status in the second year of COVID-19, in particular worsening (23%) and improvement (20%). This finding may be partly related to whether or not people were affected by coronavirus.

**Table 5:** Frequency of individual change of status in the first (rows) and second (columns) years of the pandemic for each well-being indicator (− 1=worsening, 0=unchanged, 1=improvement). Work indicator excludes upward change

	Making ends meet			Income			Health			Work			Social connections		
	− 1	0	1	−1	0	1	−1	0	1	− 1	0	1	− 1	0	1
− 1	0.09	0.08	0.01	0.32	0.01	0.00	0.03	0.04	0.02	0.02	0.05	NC	0.09	0.05	0.00
0	0.07	0.42	0.09	0.01	0.23	0.02	0.23	0.45	0.20	0.02	0.92	NC	0.01	0.81	0.02
1	0.01	0.08	0.16	0.01	0.01	0.39	0.01	0.01	0.01	NC	NC	NC	0.00	0.02	0.01

### Country evidence

**Fig. 1:**
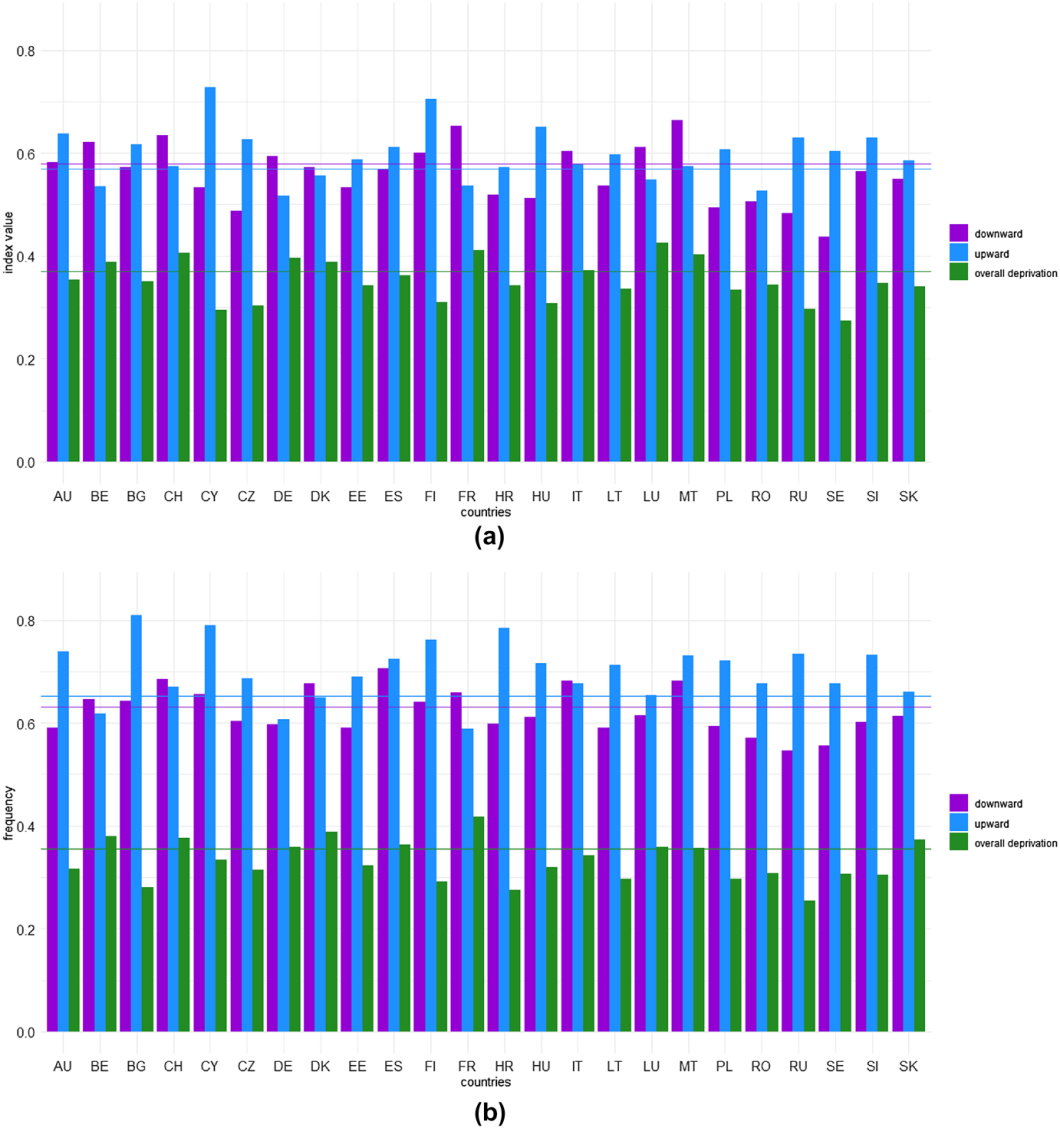
Headcount of directional well-being changes and overall deprivation by country ($\alpha =0$): first year (**a**) and second year (**b**) of the pandemic. The horizontal lines represent European averages: the purple line represents the European downward index, the light blue line the European upward index, while the green line shows the overall deprivation in Europe

**Fig. 2:**
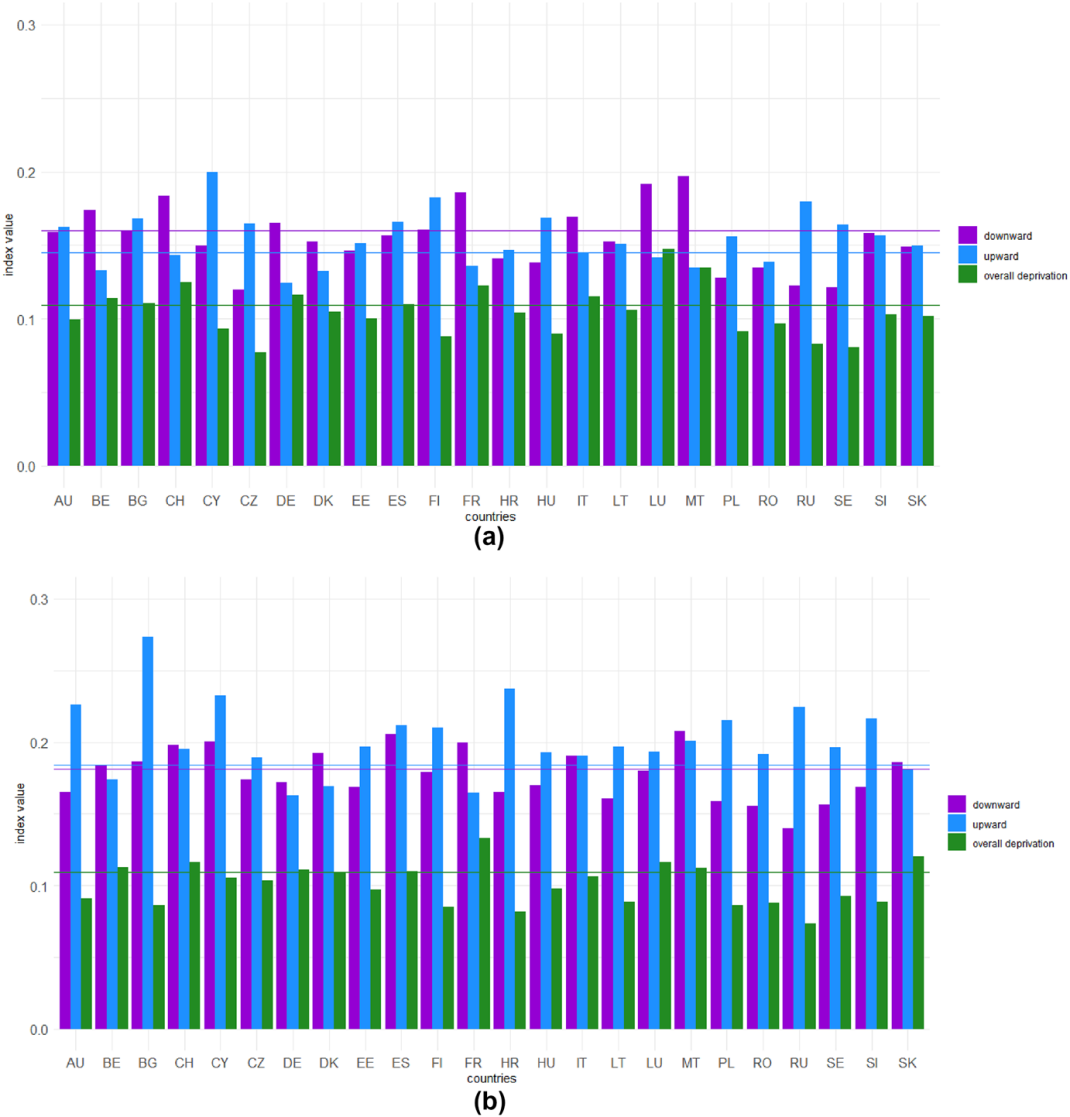
Directional change gaps and overall deprivation gap by country ($\alpha =1$): first year (**a**) and second year (**b**) of the pandemic. The horizontal lines represent European averages: the purple line represents the European downward index, the light blue line the European upward index, while the green line shows the overall deprivation in Europe

After an initial overview of the effects of COVID-19 pandemic on well-being at European level, we now aim to understand the extent to which these changes in well-being differ across European countries. Figures 1 and 2 provide graphical representations of the downward and upward changes and overall deprivation in the first and second years of the pandemic.

The European countries that suffered greater downward well-being changes (both for $\alpha =0$ and $\alpha =1$) were France, Luxembourg and Malta during the first year of the pandemic, while the Czech Republic, Spain, Italy and Malta suffered more in the second year of the pandemic.

We now want to understand (i) which well-being dimensions mainly affect the changes; (ii) whether individuals experienced changes in one or more dimensions on average; and (iii) which groups of the population were most affected by the pandemic.

**Fig. 3:**
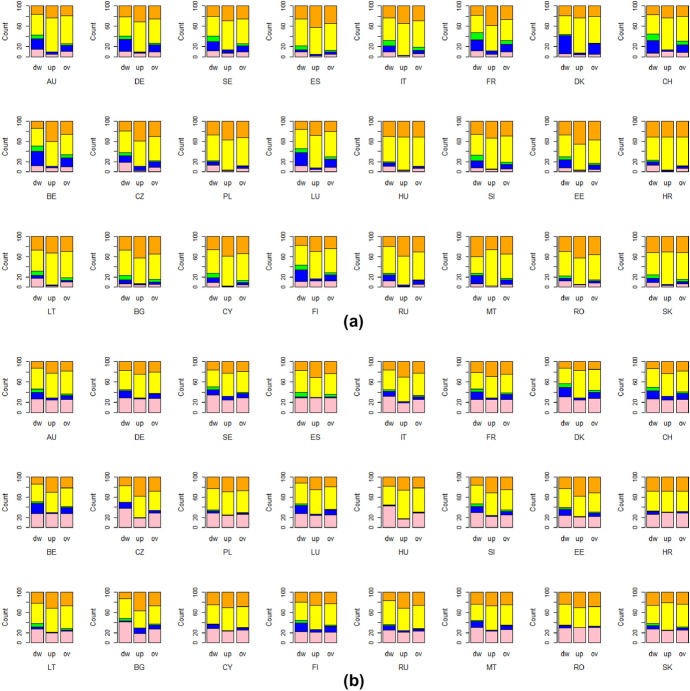
Contribution of each dimension in the well-being change indices (dw = downward, up = upward, ov = overall deprivation) by country in the first year (**a**) and the second year (**b**) of the pandemic, $\alpha =1$. Health dimension (pink), social dimension (blue), work dimension (green), ability to make ends meet (yellow), income dimension (orange)

The main drivers of the well-being changes are depicted in Fig. 3, which reveals that the most relevant dimensions are economic well-being (income and ability to make ends meet) and health. The economic dimension strongly affected overall deprivation and directional (downward/upward) well-being changes in the first period of COVID-19; some exceptions are tied to social connections for the downward changes in the Northern countries (Fig. 3a). The health dimension mainly affected well-being change in the second period of the pandemic, along with the economic dimension, as shown in Fig. 3b. Hence, it seems that in the first period of the pandemic, individuals suffered mainly due to their economic situation, while in the second year, elderly people registered relevant changes not only in their financial situation but also in their health status. Indeed the percentage of individuals whose health worsened in the second period of the analysis increased.^9^

**Fig. 4:**
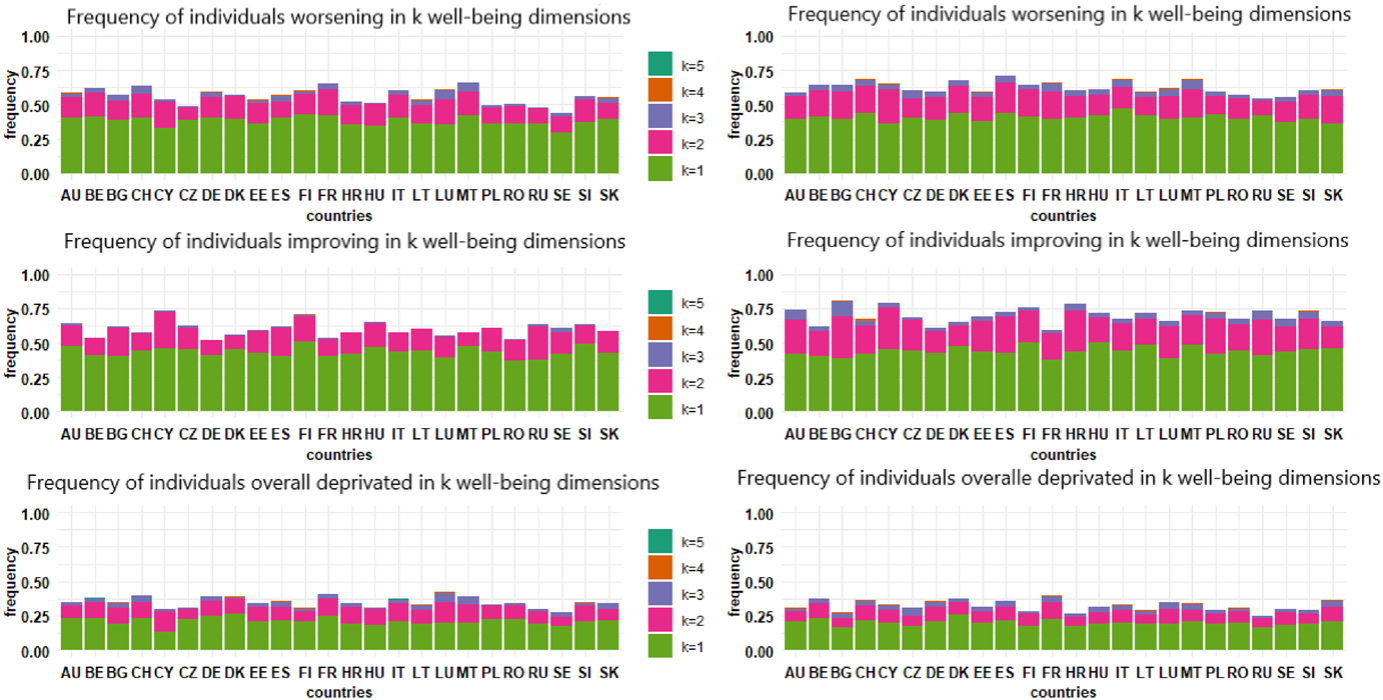
Frequency of individuals worsening, improving or being deprived in well-being indicators after one year (left panel) and two years (right panel) of the pandemic

Figure 4 shows the frequency of individuals in each country who experienced a change in one, two or more well-being indicators. In all European countries, the majority of individuals whose well-being changed as a consequence of COVID-19 registered a change only in one dimension. Almost none of the respondents declared a change in all well-being dimensions. Moving on to the second year of the pandemic, both downward and upward changes in well-being increased in comparison to the first pandemic period, revealing that the persistence of the pandemic for several months had negative effects on the well-being of European citizens.

**Fig. 5:**
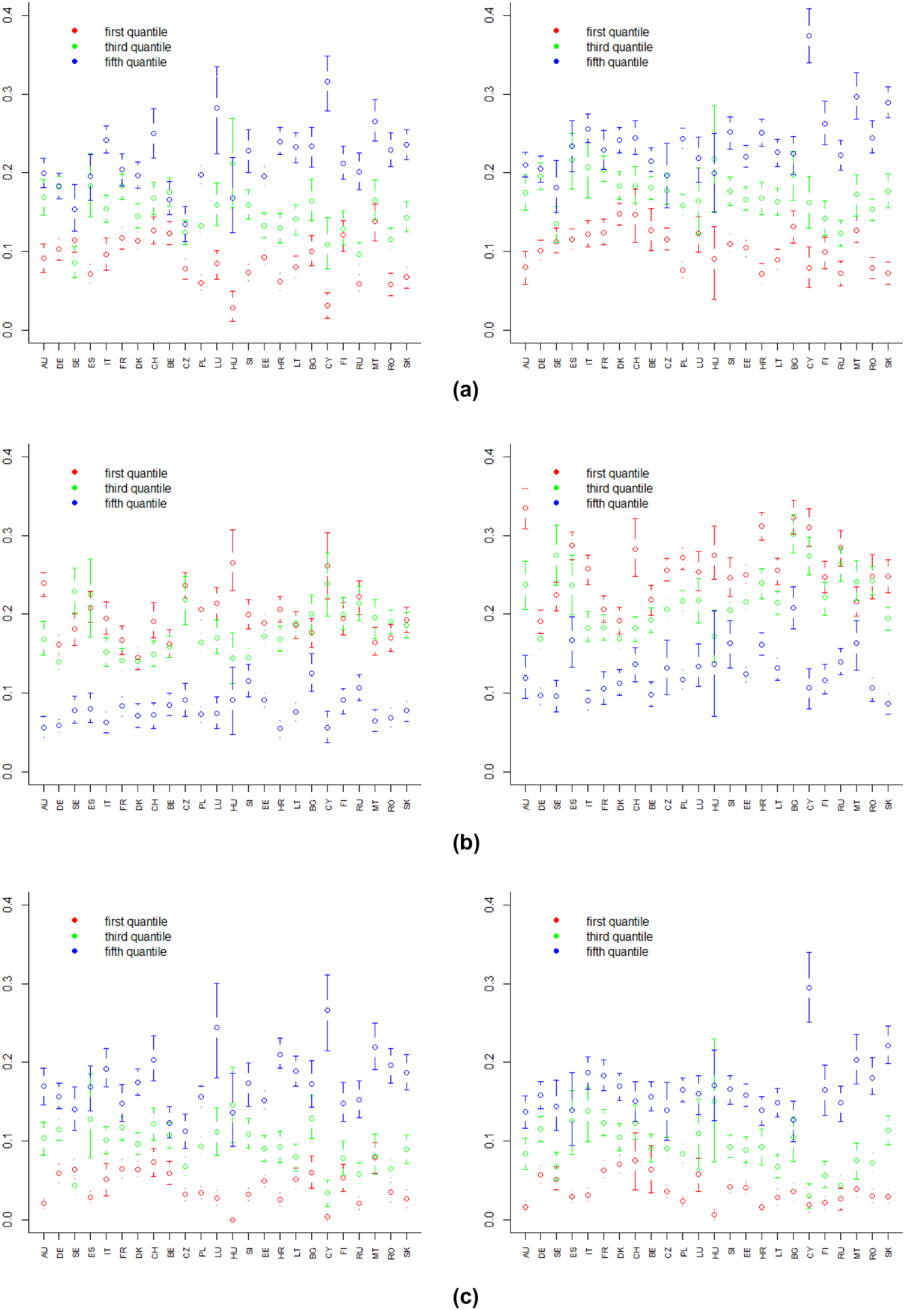
Bootstrap confidence intervals by income quintile of: downward change index (**a**), upward change index (**b**) and overall deprivation index (**c**) referring to the first year (left panel) and second year (right panel) of the pandemic, $\alpha =1$

**Fig. 6:**
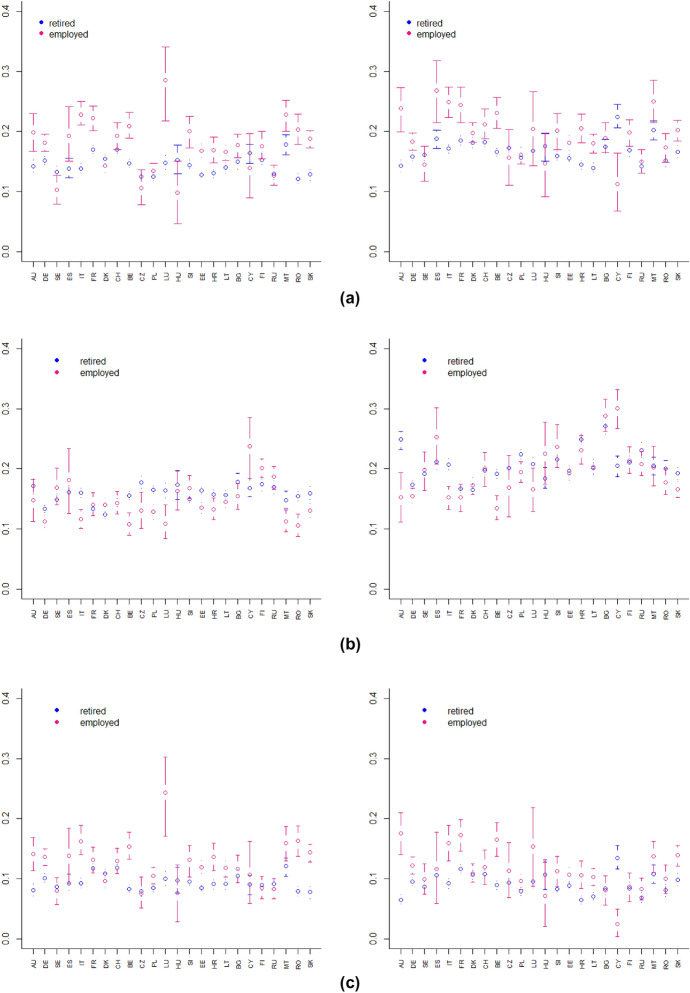
Bootstrap confidence intervals by work status of: downward change index **a**, upward change index **b** and overall deprivation index **c** referring to the first year (left panel) and second year (right panel) of the pandemic, $\alpha =1$

**Fig. 7:**
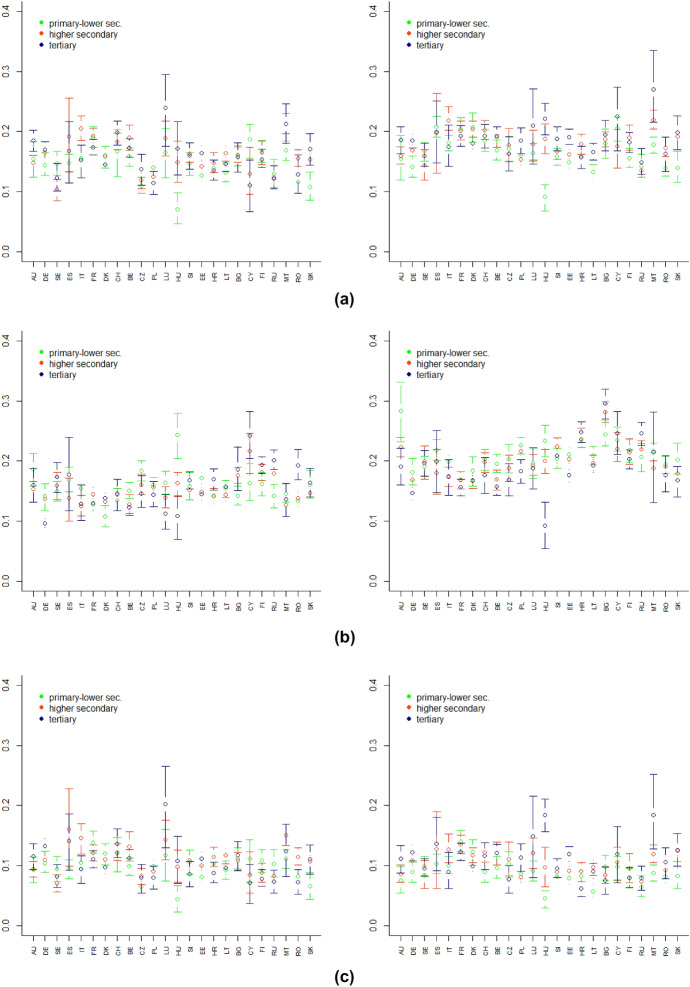
Bootstrap confidence intervals by educational level of: downward change index **a**, upward change index **a** and overall deprivation index **a** referring to the first year (left panel) and second year (right panel) of the pandemic, $\alpha =1$

Finally, for each European country we also want to understand whether there are subgroup differences. Figures 5, 6 and 7 illustrate bootstrap confidence intervals of indices of well-being change (with $\alpha =1$) by subgroup.^10^ For all countries, higher well-being losses are registered for richer individuals, since the social protection policies implemented by European countries helped the poorest people more. Employed people were also affected by the pandemic more than retired people, while the impact in terms of education was different from country to country.

## Concluding remarks

This paper proposed new indices of well-being directional changes and overall deprivation of well-being to evaluate the degree to which individual (multidimensional) well-being worsened or improved as a consequence of the COVID-19 pandemic.

The indices were obtained in two steps. First, we provided individual measures of directional changes and overall deprivation. Second, we aggregated over the entire population to obtain an index that allows for comparisons between different societies.

This paper is one of the first attempts to compare the effects of the COVID-19 pandemic on well-being among elderly people in European countries, as well as distinguishing different phases of the pandemic (first year versus second year).

The empirical application shows how the new indices contribute to disentangling the different facets of the effects of the COVID-19 pandemic on individual well-being. In particular, people with tertiary education experienced significantly stronger downward changes and fewer upward changes than people with primary or lower secondary education. Workers were significantly more deprived than retired people. Moreover, the poorest and middle classes were less affected by downward changes than individuals belonging to higher income classes. The results also highlight how the health dimension played a crucial role in the second year of the pandemic.

Future research may explore two additional directions. First, we can consider different weighting systems that allow different well-being indicators and dimensions to have a different impact on well-being changes. Second, we can perform an inter-temporal analysis and model the path of well-being changes in more than two periods of time.

## Data Availability

The SHARE data are distributed by SHARE-ERIC (Survey of Health, Ageing and Retirement in Europe-European Research Infrastructure Consortium) to registered users through the SHARE Research Data Center. Access to the data collected and generated in the SHARE projects is provided free of charge for scientific use, after individual registration.

## Footnotes

1 See http://ec.europa.eu/environment/beyond_gdp/background_en.htmlfordetails.

2 https://ec.europa.eu/eurostat/statistics-explained/index.php?title=Population_structure_and_ageing

3 Here, the parameter $\alpha$ plays the same role as $\alpha$ parameter in the Foster-Greer-Thorbecke (FGT) poverty measures (Foster et al. 1984), therefore taking only non-negative integer values. In our paper we have focused in particular on $\alpha = 0$ to replicate the headcount ratio FGT(0) and on $\alpha =1$ to recall the poverty gap FGT(1).

4 For more details, we refer to Borsch-Supan, A. *Survey of Health, Ageing and Retirement in Europe (SHARE) Wave 8. Release version: 1.0.0.* SHARE-ERIC (2021): Data set.

5 We used the OECD modified equivalence scale, which assigns a value of 1 to the household head, of 0.5 to each additional adult member and of 0.3 to each child under 14 years old.

6 Income and ability to make ends meet are collected at household level, and we have assigned them to each household member since our analysis is carried out at individual level.

7 In the Wave 9 Corona Survey, the health dimension compares self-perceived health: the change in health dimension is equal to -1,0 or 1 if the respondent affirms a change for the worse in his/her health.

8 Outliers were removed using a trimming technique for incomes above the 99th percentile.

9 For sake of completeness, we have also analyzed the sensitivity of our measure of well-being changes to different weights’ systems. Since we have noted that the health dimension plays a crucial role especially in the second year of the pandemic, we have tested also two alternative systems of weights adopting a “health-oriented” approach as in Osberg and Sharpe (2002). In particular, we have assigned more weight to the health dimension while keeping equal weights for the other domains, specifically 0.4 in the first case and 0.6 in the second case. Looking at the rankings of countries, we tested the difference with respect to the equal weight approach. Kendall non-parametric test rejected, in both cases, the null hypothesis of independence.

10 According to the parameters of the bootstrap procedure, we used 100 replicates and the size of the sample in each replication is the $70\%$ of the original sample.
